# Machine learning for early discrimination between transient and persistent acute kidney injury in critically ill patients with sepsis

**DOI:** 10.1038/s41598-021-99840-6

**Published:** 2021-10-12

**Authors:** Xiao-Qin Luo, Ping Yan, Ning-Ya Zhang, Bei Luo, Mei Wang, Ying-Hao Deng, Ting Wu, Xi Wu, Qian Liu, Hong-Shen Wang, Lin Wang, Yi-Xin Kang, Shao-Bin Duan

**Affiliations:** 1 Department of Nephrology, Hunan Key Laboratory of Kidney Disease and Blood Purification, The Second Xiangya Hospital of Central South University, 139 Renmin Road, Changsha, 410011 Hunan China; 2 Information Center, The Second Xiangya Hospital of Central South University, Changsha, 410011 Hunan China; 3 Department of Information Systems, City University of Hong Kong, Tat Chee Avenue, Kowloon, 999077 Hong Kong SAR China

**Keywords:** Infectious diseases, Kidney diseases, Nephrology, Kidney, Kidney diseases, Renal replacement therapy, Outcomes research, Risk factors, Prognosis

## Abstract

Acute kidney injury (AKI) is commonly present in critically ill patients with sepsis. Early prediction of short-term reversibility of AKI is beneficial to risk stratification and clinical treatment decision. The study sought to use machine learning methods to discriminate between transient and persistent sepsis-associated AKI. Septic patients who developed AKI within the first 48 h after ICU admission were identified from the Medical Information Mart for Intensive Care III database. AKI was classified as transient or persistent according to the Acute Disease Quality Initiative workgroup consensus. Five prediction models using logistic regression, random forest, support vector machine, artificial neural network and extreme gradient boosting were constructed, and their performance was evaluated by out-of-sample testing. A simplified risk prediction model was also derived based on logistic regression and features selected by machine learning algorithms. A total of 5984 septic patients with AKI were included, 3805 (63.6%) of whom developed persistent AKI. The artificial neural network and logistic regression models achieved the highest area under the receiver operating characteristic curve (AUC) among the five machine learning models (0.76, 95% confidence interval [CI] 0.74–0.78). The simplified 14-variable model showed adequate discrimination, with the AUC being 0.76 (95% CI 0.73–0.78). At the optimal cutoff of 0.63, the sensitivity and specificity of the simplified model were 63% and 76% respectively. In conclusion, a machine learning-based simplified prediction model including routine clinical variables could be used to differentiate between transient and persistent AKI in critically ill septic patients. An easy-to-use risk calculator can promote its widespread application in daily clinical practice.

## Introduction

Acute kidney injury (AKI) is a common and severe complication in critically ill patients, especially in patients with sepsis^1,2^. The complex condition in which patients meet consensus criteria for sepsis and AKI simultaneously is recognized as sepsis-associated AKI (SA-AKI), which is associated with significantly higher risks of mortality and chronic renal insufficiency^3–5^. Up to now, the prophylactic and therapeutic options for SA-AKI are still limited. Both severity and duration of SA-AKI can affect short- and long-term adverse outcomes.

Most recently, the Acute Disease Quality Initiative (ADQI) 16 Workgroup suggested that AKI be classified as transient (a complete reversal of AKI within 48 h) or persistent (the continuance of AKI beyond 48 h)^6^. Compared to transient AKI, persistent AKI is related to enhanced and sustained host response dysregulation and adverse consequences in critically ill septic patients^7,8^. Early recognition of persistent AKI is significant for risk stratification and individualized therapy, such as fluid management and the use of renal replacement therapy (RRT)^6,9^. However, since complex mechanisms including microcirculatory dysfunction and inflammatory response may co-exist in the pathophysiology of SA-AKI, traditional indicators for renal blood flow have been reported to play a limited role in differentiating between transient and persistent AKI^10–13^. Additionally, a few studies assessing the predictive value of function or damage biomarkers for persistent AKI have suggested that most biomarkers showed poor performance while the others need further clinical validation^14–17^. At present, there is a lack of clinical information on how to identify patients who are likely to develop persistent AKI.

The development of machine learning algorithms may provide an opportunity for early prediction of persistent AKI by integration of a large quantity of data from electronic health records, such as demographics, diagnoses, routinely collected measurements and interventions. These advanced data-driven approaches can deal with high-dimension data, fit complex relationships and identify important variables associated with the outcome. They outperform conventional modeling methods which require the independence between predictors and include variables selected mainly according to their statistical significance or known clinical relevance. Machine learning has been applied in the biomedical domain, such as disease diagnosis, outcome prediction, medical image analysis and treatment^18–21^.

The primary objective of this study was to use machine learning methods to develop a prediction model for the persistence of SA-AKI in an attempt to identify patients at high risk of persistent AKI in daily clinical practice.

## Methods

### Source of data

Data were extracted from the Medical Information Mart for Intensive Care III (MIMIC-III) database v1.4^22^. MIMIC-III is a large and openly accessible database comprising electronic health records of 61,532 intensive care unit (ICU) stays from the Beth Israel Deaconess Medical Center (BIDMC, Boston, MA) between 2001 and 2012. The database was approved by the Institutional Review Boards of BIDMC and Massachusetts Institute of Technology and informed consent was waived by them because all patient identifiers in the database were removed. One of the authors has completed the required training course and obtained access to the database (certification number: 40010711). The study was performed in accordance with the Declaration of Helsinki.

### Study population

This study included adult patients who were admitted to ICU with sepsis and developed AKI within the first 48 h of the ICU stay. Sepsis was defined based on the updated Sepsis-3 criteria as suspected infection (the concomitant administration of antibiotics and sampling of body fluid culture) with the Sequential Organ Failure Assessment (SOFA) score ≥ 2 points^23,24^. Patients with suspicion of infection more than 24 h before or after ICU admission were excluded. The microbiology information was extracted to verify the locations and pathogens of positive cultures taken during the suspected infection time. SOFA score was calculated using data within the first 24 h after ICU admission. AKI was diagnosed and staged according to the Kidney Disease: Improving Global Outcomes (KDIGO) guideline using both serum creatinine (SCr) and urine output (UO) criteria^25^. Baseline SCr was defined as the lowest SCr value during 7 days before ICU admission^26,27^. For patients without available pre-admission SCr, we used the first SCr measurement after ICU admission as the baseline SCr^26^. UO rate was calculated by dividing the volume of UO into 6-h, 12-h and 24-h time periods. We analyzed only the first ICU stay for patients who were admitted to ICU more than once. We also excluded patients with age < 18 years old, end-stage renal disease, ICU stay < 48 h, non-AKI and missing data for AKI during the first 48 h.

### Outcomes

The primary outcome was the persistence of AKI, which was determined in accordance with the ADQI 16 workgroup consensus^6^. Transient AKI was defined as reversal of AKI within 48 h after AKI diagnosis and for at least 48 h. In contrast, AKI was considered persistent if AKI criteria or RRT use remained present beyond 48 h after AKI diagnosis, or if the condition reversed within 48 h but relapsed within the next 48 h^6,7^. Patients with follow-up time < 48 h or missing data for the persistence of AKI were excluded from the analysis. Secondary outcomes included 28-day mortality, 90-day mortality and use of RRT within 28 days after ICU admission.

### Data extraction

We obtained demographic and clinical data within the first 48 h after ICU admission using PostgreSQL tools (version 9.6.20) and Navicat Premium (version 15.0.12). Comorbidities and diagnoses were identified based on the recorded International Classification of Diseases 9th Edition code. Vital signs including temperature, heart rate, respiratory rate and mean arterial pressure were extracted from the electronic charted data. Laboratory data including hemoglobin, white blood cell count, platelet count, bilirubin, albumin, arterial pH, partial pressure of oxygen, partial pressure of carbon dioxide, anion gap, serum electrolytes (sodium, potassium, chloride and bicarbonate), lactate, international normalized ratio and partial thromboplastin time were also recorded. We used the values related to the greatest disease severity for variables measured more than once during the first 48 h. Accordingly, both the maximum and minimum values of some variables were included. In addition, the use of mechanical ventilation, vasopressors, diuretics and RRT and the volume of mean daily intravenous infusion within the first 48 h were collected. We left out RRT initiation when determining the AKI stage, as we chose to record it as another variable.

### Statistical analysis

Baseline characteristics and outcomes were compared between patients with transient and persistent AKI. Continuous variables were presented as medians (with interquartile ranges) and compared using Mann–Whitney U test. Categorical variables were presented as numbers (with percentages) and compared using chi-square tests. To ensure the facticity and reliability of the prediction model, we removed two variables with > 30% missing data from model construction, namely maximum bilirubin and minimum albumin (see Supplementary Table S1 online). Random forest (RF) method was used to impute missing values in variables with ≤ 30% observations missing (R package missForest, version 1.4). Supplementary Table S2 online lists all 44 candidate predictors included for application to machine learning.

The sample was randomly divided into the training and testing set by the ratio of 7 to 3. Five machine learning algorithms were used to develop prediction models for persistent AKI in the training set, including logistic regression, RF, support vector machine (SVM), artificial neural network (ANN) and extreme gradient boosting (XGB). RF is a tree-based algorithm, which integrates multiple decision trees through majority voting to determine the results of classification^28^. Gini index was used as the criteria for impurity measurement during the training process. SVM is a supervised classifier, the purpose of which is to establish the optimal maximum-margin hyperplane as decision boundary^29^. We chose Gaussian kernel function as the kernel when developing the SVM model. ANN is a mathematical model simulating the structure and function of biological neural networks, which contains connected nodes named artificial neurons and multiple layers (typically input layer, hidden layer and output layer)^30^. XGB is also a tree-based ensemble classifier, which obtains the final output by weight of multiple weak learners (decision trees) and gradient descent algorithm for minimizing the loss function^31^. Before model construction, categorical variables were preprocessed by one-hot encoding and the prediction variables were standardized. For each machine learning algorithm, we firstly set default hyper-parameters to establish an initial model. After that, parameter tuning was performed by manual grid search. We used five-fold cross-validation to identify optimal hyper-parameters and avoid over-fitting. Briefly, the training set was randomly divided into 5 roughly equal-sized subsets, and then 4 of them were fit into the model while the other was used for model validation. This process was repeated 5 times so that every subset could serve as a validation set. Subsequently, the performance of the final model was assessed on the testing set. We calculated several evaluation indexes of each model, including the area under the receiver operating characteristic curve (AUC), accuracy, precision, recall and F1 score. AUC was selected as the primary performance metric, which was considered an ideal evaluation metric for classifiers independent of threshold setting.

To further extend the clinical applicability of machine learning methods, we also developed a risk prediction model by simplifying the input variables. Firstly, all features were sorted by XGB according to their contribution to each tree in the learning process, and the top 20 important features were selected^31^. Then we used least absolute shrinkage and selection operator (LASSO) method for further feature selection^32^. During the process, cross-validation was performed and the value of λ was identified according to the most regularized model, in which the cross-validated error is within one standard error of the minimum. Fourteen variables were selected as predictors of persistent AKI. Finally, logistic regression was used to construct the simplified prediction model. Model performance was evaluated in the testing set, with the optimal cutoff identified by the maximum Youden index in the training set.

Statistical analyses were conducted using R 4.0.4 (https://cran.r-project.org) and Python 3.8 (https://www.python.org). *P* value < 0.05 was considered statistically significant.

## Results

### Patient characteristics

**Figure 1:**
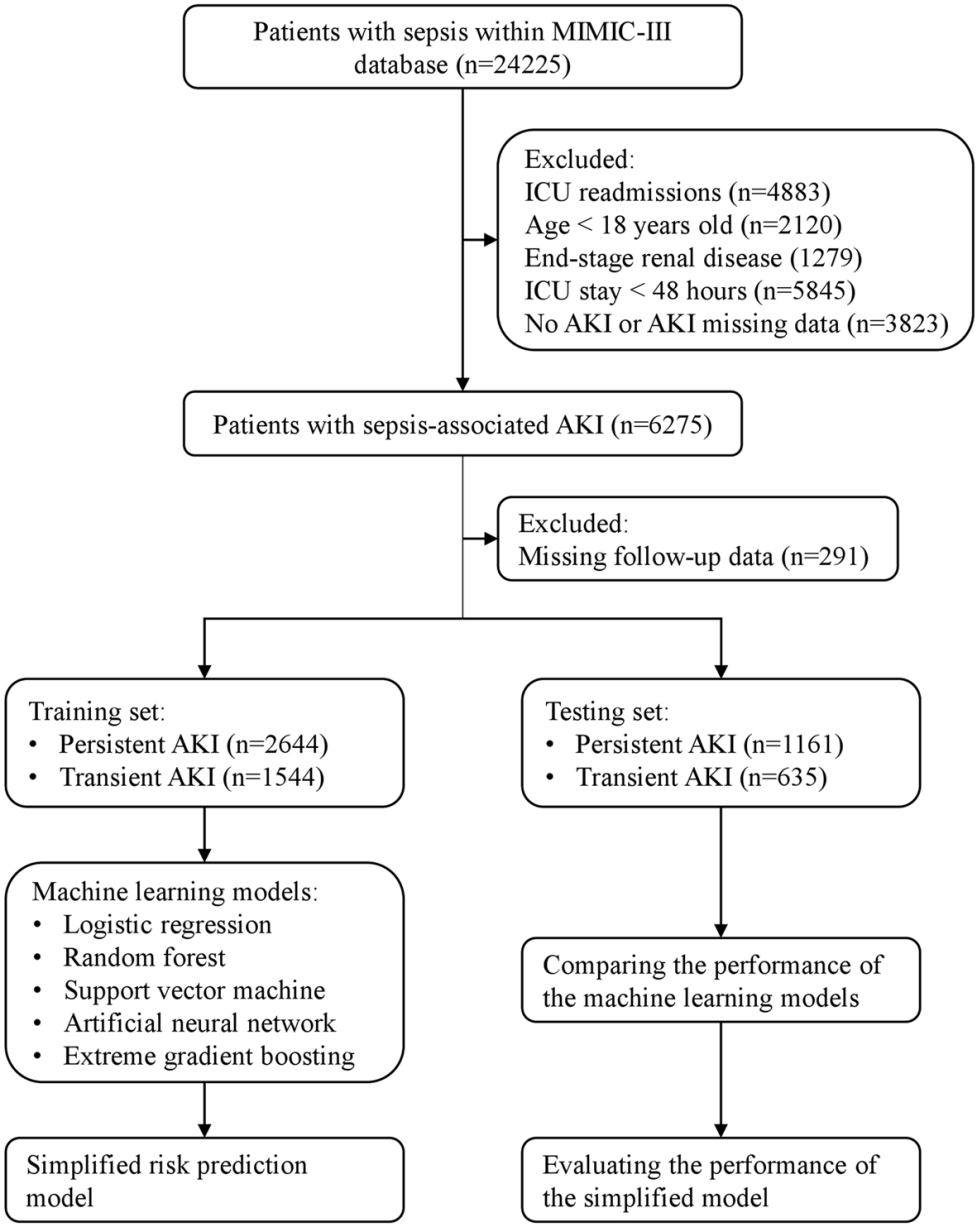
Flow diagram of patient selection, model establishment and internal validation. MIMIC-III, Medical Information Mart for Intensive Care III; ICU, intensive care unit; AKI, acute kidney injury.

A total of 5984 SA-AKI patients were enrolled in our study from 24,225 septic patients admitted to ICU during the study period. Among them, 2179 (36.4%) patients had an early complete reversal and 3805 (63.6%) developed persistent AKI (Fig. 1).

Baseline characteristics and outcomes of patients stratified by the persistence of AKI are shown in Table 1. Compared to patients with transient AKI, patients with persistent AKI had a higher proportion of emergency admission and medical ICU stay. The prevalence of diabetes mellitus, congestive heart failure, liver disease and chronic kidney disease (CKD) were higher in the persistent AKI patients. Most of the vital signs and laboratory data differed significantly between the two groups, and the measurements were mainly associated with higher disease severity in the persistent AKI group. Furthermore, a larger percentage of the persistent AKI patients received mechanical ventilation, vasopressors and RRT during the first 48 h. Renal dysfunction was more severe in the persistent AKI group, as reflected by higher AKI stage according to SCr or UO criteria. The locations and pathogens of microbiology cultures in SA-AKI patients are shown in Supplementary Tables S3, S4 online, and the 20 most common diagnoses in SA-AKI patients are shown in Supplementary Table S5 online.

**Table 1:** Baseline characteristics and outcomes of patients stratified by the persistence of AKI.

Variables	Transient AKI (n = 2179)	Persistent AKI (n = 3805)	*P* value
Age (year)	68 (56–79)	69 (57–80)	0.12
Sex, male, n (%)	1210 (55.5)	2145 (56.4)	0.55
Ethnicity, n (%)			0.74
White	1616 (74.2)	2849 (74.9)	
Black	158 (7.3)	279 (7.3)	
Other	405 (18.6)	677 (17.8)	
ICU type, n (%)			0.002
MICU	811 (37.2)	1592 (41.8)	
SICU/TSICU	659 (30.2)	1039 (27.3)	
CCU/CSRU	709 (32.5)	1174 (30.9)	
Admission type, n (%)			< 0.001
Elective	373 (17.1)	488 (12.8)	
Emergency	1767 (81.1)	3222 (84.7)	
Urgent	39 (1.8)	95 (2.5)	
**Comorbidities, n (%)**			
Hypertension	1224 (56.2)	2073 (54.5)	0.22
Diabetes mellitus	559 (25.7)	1226 (32.2)	< 0.001
Congestive heart failure	691 (31.7)	1558 (40.9)	< 0.001
Peripheral vascular disease	283 (13.0)	534 (14.0)	0.27
Chronic pulmonary disease	468 (21.5)	843 (22.2)	0.56
Liver disease	163 (7.5)	496 (13.0)	< 0.001
AIDS	16 (0.7)	41 (1.1)	0.24
Metastatic cancer	115 (5.3)	228 (6.0)	0.28
Chronic kidney disease	227 (10.4)	578 (15.2)	< 0.001
**Vital signs**			
Minimum temperature (℃)	36.0 (35.6–36.4)	35.9 (35.5–36.4)	< 0.001
Maximum temperature (℃)	37.9 (37.3–38.4)	37.8 (37.3–38.4)	0.10
Maximum heart rate (bpm)	110 (98–126)	114 (99–129)	< 0.001
Maximum respiratory rate (bpm)	30 (26–34)	30 (26–35)	0.001
Minimum MAP (mmHg)	54 (48–60)	52 (47–59)	< 0.001
**Laboratory data**			
Minimum hemoglobin (g/dL)	9.3 (8.2–10.5)	9.1 (8.1–10.4)	0.003
Minimum WBC (× 10^9^/L)	9.6 (7.0–12.7)	9.7 (6.9–13.2)	0.90
Maximum WBC (× 10^9^/L)	14.1 (10.6–18.6)	14.5 (10.5–19.6)	0.19
Minimum platelet (× 10^9^/L)	160 (109–224)	147 (94–220)	< 0.001
Maximum bilirubin (mg/dL)	0.8 (0.5–1.7)	1.0 (0.5–3.0)	< 0.001
Minimum albumin (g/dL)	2.8 (2.4–3.2)	2.7 (2.3–3.2)	< 0.001
Minimum pH	7.33 (7.27–7.38)	7.30 (7.23–7.37)	< 0.001
Minimum PaO_2_ (mmHg)	82 (68–106)	75 (63–94)	< 0.001
Minimum PaCO_2_ (mmHg)	34 (30–39)	33 (29–38)	< 0.001
Maximum PaCO_2_ (mmHg)	46 (41–53)	47 (41–55)	0.001
Maximum anion gap (mmol/L)	14 (12–17)	16 (13–19)	< 0.001
Minimum sodium (mmol/L)	136 (133–139)	136 (133–139)	0.48
Maximum sodium (mmol/L)	141 (138–143)	141 (138–143)	0.40
Maximum potassium (mmol/L)	4.5 (4.1–5.0)	4.6 (4.2–5.2)	< 0.001
Minimum chloride (mmol/L)	103 (100–107)	103 (99–106)	< 0.001
Maximum chloride (mmol/L)	109 (106–112)	109 (105–112)	< 0.001
Minimum bicarbonate (mmol/L)	22 (19–24)	21 (18–24)	< 0.001
Maximum lactate (mmol/L)	2.2 (1.5–3.4)	2.5 (1.6–4.3)	< 0.001
Maximum INR	1.4 (1.2–1.6)	1.5 (1.3–1.9)	< 0.001
Maximum PTT (sec)	35.0 (29.1–46.8)	38.7 (30.8–59.7)	< 0.001
**Interventions**			
Mechanical ventilation, n (%)	1587 (72.8)	2938 (77.2)	< 0.001
Vasopressors, n (%)	1062 (48.7)	2152 (56.6)	< 0.001
RRT initiation, n (%)	10 (0.5)	247 (6.5)	< 0.001
Diuretics, n (%)	1145 (52.5)	2035 (53.5)	0.50
Daily fluid infusion (mL)	2922 (1916–4199)	3194 (1958–4840)	< 0.001
AKI stage by SCr criteria, n (%)			< 0.001
1	543 (24.9)	1242 (32.6)	
2	46 (2.1)	359 (9.4)	
3	28 (1.3)	444 (11.7)	
AKI stage by UO criteria, n (%)			< 0.001
1	528 (24.2)	379 (10.0)	
2	1147 (52.6)	1815 (47.7)	
3	167 (7.7)	1172 (30.8)	
**Outcomes, n (%)**			
RRT use	18 (0.8)	463 (12.2)	< 0.001
28–day mortality	238 (10.9)	992 (26.1)	< 0.001
90–day mortality	374 (17.2)	1330 (35.0)	< 0.001

AKI, acute kidney injury; ICU, intensive care unit; MICU, medical intensive care unit; SICU, surgical intensive care unit; TSICU, trauma surgical intensive care unit; CCU, coronary care unit; CSRU, cardiac surgery recovery unit; AIDS, acquired immune deficiency syndrome; MAP, mean arterial pressure; WBC, white blood cell; PaO_2_, partial pressure of oxygen; PaCO_2_, partial pressure of carbon dioxide; INR, international normalized ratio; PTT, partial thromboplastin time; RRT, renal replacement therapy; SCr, serum creatinine; UO, urine output.

Continuous variables were presented as median (interquartile range) and categorical variables were presented as n (%).

### Prediction models using machine learning algorithms

**Figure 2:**
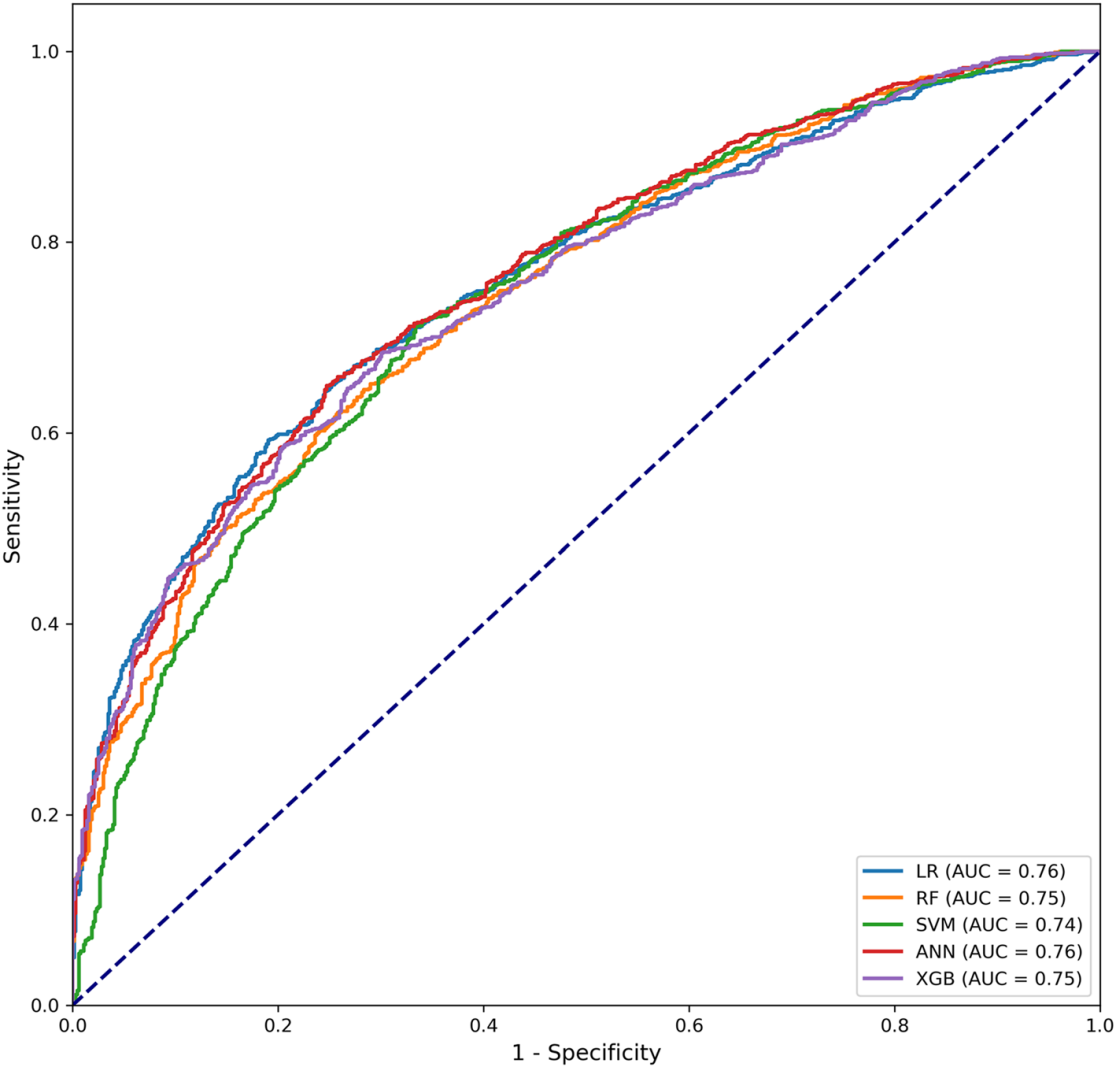
Receiver operating characteristic curves of the machine learning models in the testing set. LR, logistic regression; RF, random forest; SVM, support vector machine; ANN, artificial neural network; XGB, extreme gradient boosting; AUC, area under the receiver operating characteristic curve.

We randomly allocated 70% of SA-AKI patients to the training set and the remaining 30% to the testing set. Baseline characteristics were not significantly different between the training and testing set (see Supplementary Table S6 online). Among the five machine learning models, the ANN model and the logistic regression model exhibited the highest AUC (0.76, 95% confidence interval [CI] 0.74–0.78) in the testing set (Table 2, Fig. 2). The ANN model achieved the highest accuracy of 0.71. Moreover, the XGB model showed the highest recall of 0.81, while the RF model showed the highest precision and F1 score of 0.89 and 0.80 respectively (Table 2).

**Table 2:** Performance comparison of the machine learning models in the testing set.

Models	AUC (95% CI)	Accuracy	Precision	Recall	F1 score
Logistic regression	0.76 (0.74–0.78)	0.70	0.80	0.75	0.78
Random forest	0.75 (0.72–0.77)	0.70	0.89	0.72	0.80
Support vector machine	0.74 (0.72–0.76)	0.70	0.83	0.74	0.78
Artificial neural network	0.76 (0.74–0.78)	0.71	0.80	0.76	0.78
Extreme gradient boosting	0.75 (0.73–0.77)	0.66	0.62	0.81	0.70

AUC, area under the receiver operating characteristic curve; CI, confidence interval.

### Simplified risk prediction model

**Figure 3:**
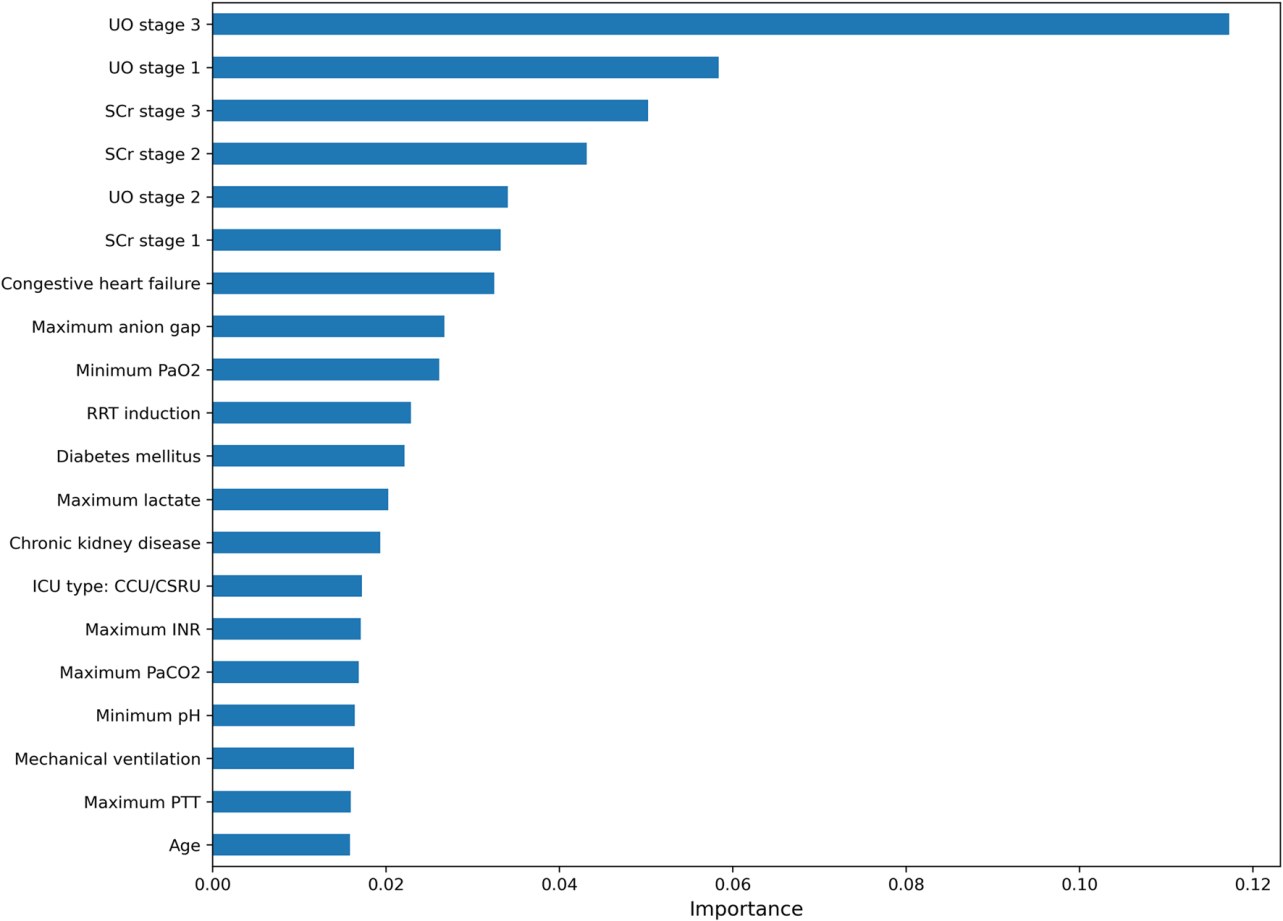
The top 20 important features derived from the XGB model. UO, urine output; SCr, serum creatinine; PaO_2_, partial pressure of oxygen; RRT, renal replacement therapy; ICU, intensive care unit; CCU, coronary care unit; CSRU, cardiac surgery recovery unit; INR, international normalized ratio; PaCO_2_, partial pressure of carbon dioxide; PTT, partial thromboplastin time.

**Figure 4:**
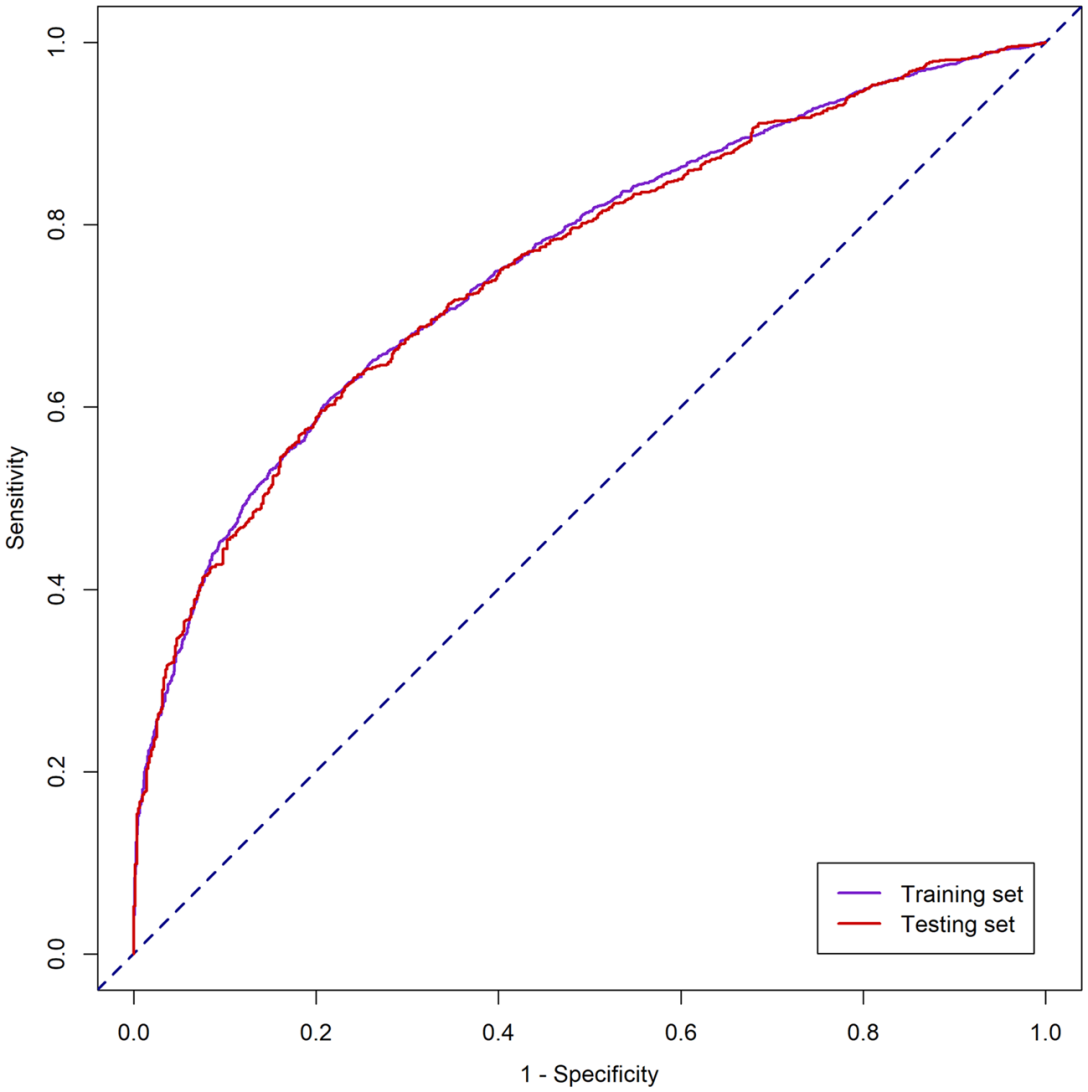
Receiver operating characteristic curve of the simplified risk prediction model in the training and testing set.

**Figure 5:**
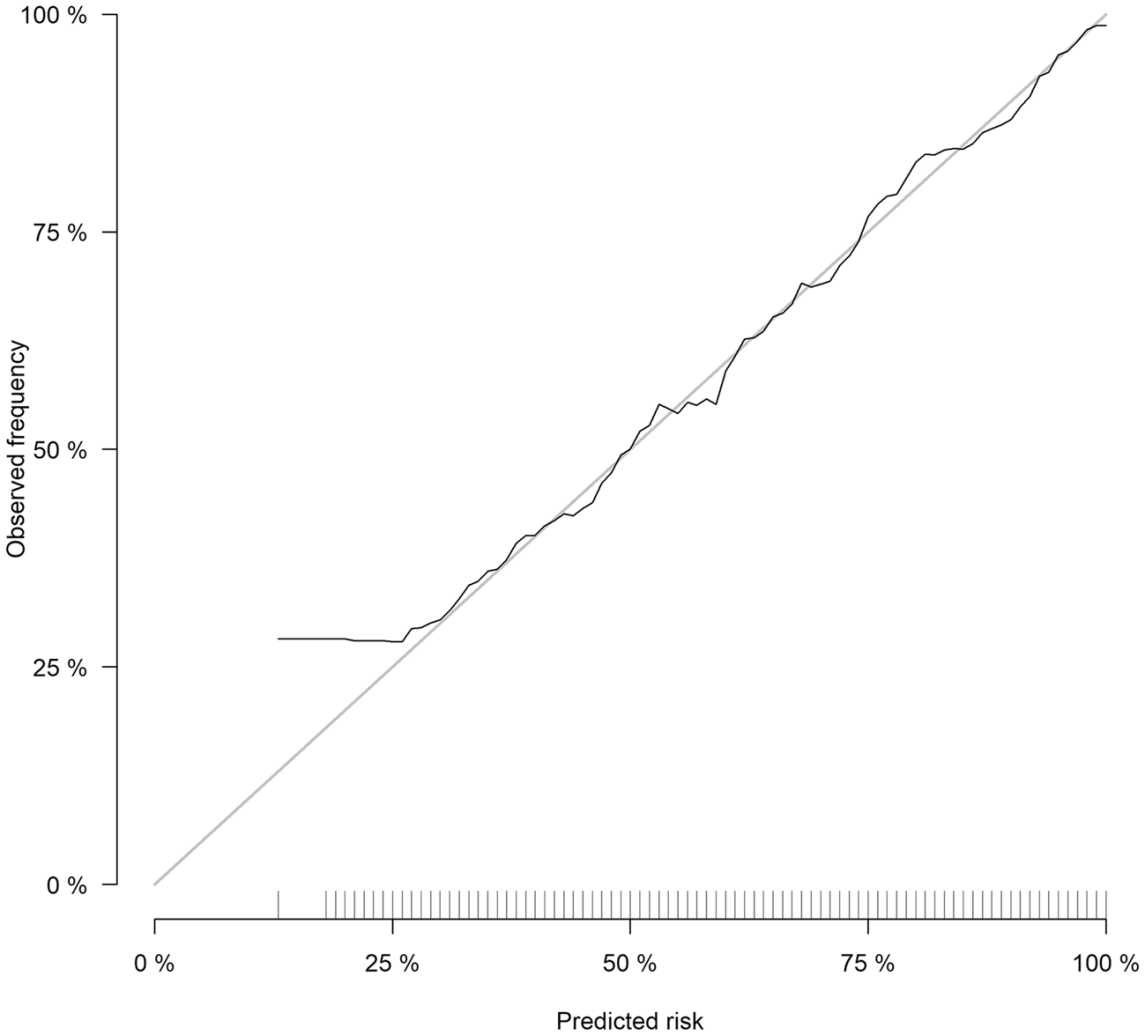
Calibration curve of the simplified risk prediction model in the training set. The Brier score of the model was 0.189 (95% confidence interval 0.184–0.194).

The simplified risk prediction model was established based on the features selected by XGB and LASSO algorithms. The top 20 important features derived from the XGB model are shown in Fig. 3. Ultimately, fourteen variables were selected and entered into the logistic regression model (Table 3). The simplified model showed adequate discrimination, with an AUC of 0.76 (95% CI 0.74–0.77) in the training set and 0.76 (95% CI 0.73–0.78) in the testing set (Fig. 4). The calibration of the model was overall good, except that it underestimated the risk of persistent AKI when the observed frequency was relatively low (Fig. 5). At the optimal cutoff of 0.63, the simplified model achieved a sensitivity of 63%, specificity of 76%, positive predictive value of 83% and negative predictive value of 53% in the testing set (Table 4).

**Table 3:** Simplified risk prediction model for persistent AKI.

Variables	Coefficient	CI		*P* value
		2.5%	97.5%	
Age	0.0062	0.0015	0.0108	0.009
Diabetes mellitus	0.2597	0.1012	0.4189	0.001
Congestive heart failure	0.3208	0.1650	0.4771	< 0.001
Chronic kidney disease	0.1475	− 0.0764	0.3740	0.20
Minimum PaO_2_	− 0.0021	− 0.0041	− 0.0001	0.038
Maximum PaCO_2_	0.0093	0.0028	0.0159	0.005
Maximum anion gap	0.0261	0.0039	0.0484	0.021
Maximum lactate	0.0209	− 0.0214	0.0640	0.34
Maximum INR	0.0690	− 0.0067	0.1505	0.09
Maximum PTT	0.0027	0.0003	0.0051	0.030
Mechanical ventilation	0.2707	0.0948	0.4468	0.003
RRT initiation	1.3618	0.6010	2.2734	0.001
**AKI stage by SCr criteria**				
1	0.8567	0.6653	1.0511	< 0.001
2	2.3339	1.9111	2.7885	< 0.001
3	2.5851	2.0773	3.1443	< 0.001
**AKI stage by UO criteria**				
1	0.4943	0.2012	0.7887	< 0.001
2	1.2795	1.0217	1.5397	< 0.001
3	2.1690	1.8653	2.4789	< 0.001

AKI, acute kidney injury; CI, confidence interval; PaO_2_, partial pressure of oxygen; PaCO_2_, partial pressure of carbon dioxide; INR, international normalized ratio; PTT, partial thromboplastin time; RRT, renal replacement therapy; SCr, serum creatinine; UO, urine output.

**Table 4:** Performance of the simplified risk prediction model in the training and testing set.

Performance metrics	Training set	Testing set
Cutoff value	0.63	0.63
AUC (95% CI)	0.76 (0.74–0.77)	0.76 (0.73–0.78)
Sensitivity	0.61	0.63
Specificity	0.78	0.76
PPV	0.83	0.83
NPV	0.54	0.53

AUC, area under the receiver operating characteristic curve; CI, confidence interval; PPV, positive predictive value; NPV, negative predictive value.

We used Matlab software (version 9.2) to establish a risk calculator, which could be applied to automatically compute the risk of persistent AKI for SA-AKI patients in clinical settings (see Supplementary Fig. S1 online).

## Discussion

In the present study, we explored the applicability of machine learning methods to differentiate between transient and persistent AKI in a large population of SA-AKI patients. The ANN and logistic regression models exhibited the highest AUC among the five machine learning models. Additionally, a simplified risk prediction model was proposed, based on the combination of machine learning algorithms and logistic regression, and could be easily implemented using the risk calculator in daily routines.

A growing body of evidence suggests that duration of AKI or renal recovery is associated with outcomes in critically ill septic patients^2,7,8,33,34^. Several clinical tools, including urinary indices^10–12^, imaging techniques^13,17^, prediction models^35,36^, and biomarkers^14–17^, were investigated in previous studies to predict renal recovery or its surrogate, namely progression to severe AKI. Nevertheless, they were found to be poorly effective or have not been validated in patients with sepsis^9^. A recent study enrolling 184 septic shock patients with AKI found a poor performance of urine cell cycle arrest biomarkers for predicting persistent AKI, with an AUC of 0.67 (95% CI 0.59–0.73). Of note, they also proposed a prediction model combining SCr, UO, norepinephrine dose and extrarenal SOFA at baseline, which performed well with an AUC of 0.81 (95% CI 0.74–0.86)^16^. Due to the complexity of SA-AKI, the clinical model integrating routine parameters may be more effective for predicting short-term reversibility of AKI than any parameter considered alone. A possible way to achieve this is to utilize advanced machine learning approaches, which have been applied in the prevention and management of AKI, such as predicting the development of AKI^37–41^, volume responsiveness in patients with oliguria^42^ and mortality in critically ill AKI patients^43–45^. Our study corroborated the promise indicated by these previous studies and extended them by demonstrating the applicability of machine learning methods for predicting persistent AKI in a large cohort of SA-AKI patients.

In the current study, ANN and logistic regression achieved the highest AUC among the five machine learning methods. Compared with traditional modeling methods, ANN has the advantages of strong nonlinear mapping ability, great adaptability and high fault tolerance. Several recent studies have shown the effectiveness of neural network-based models in predicting the development of AKI. Le et al. proposed a convolutional neural networks prediction system, which outperformed the XGB model and the SOFA score in predicting AKI 48 h before onset in ICU patients^40^. Similarly, Kim et al. used recurrent neural network to assess future AKI occurrence and individualized AKI risk factors in real time among hospitalized patients^41^. Hofer et al. applied the deep neural networks to create models for postoperative AKI, mortality, reintubation, and the combined outcome, which exhibited superior performance to the ASA score^46^. However, due to its “black box” characteristic, ANN is also hard to calculate and interpret. It is difficult to exhibit the complex association between different layers and nodes intuitively and to explain the exact impact of each input variable on the final result, which may limit its rapid clinical application. In this study, the conventional logistic regression showed higher AUC than several novel machine learning algorithms. The results were mainly determined by the nature of the dataset, as any specific modeling approach could not be the optimal method for all tasks^47^. In the logistic regression model, each variable’s influence on outcome can be directly reflected by the regression coefficient. Hence, we further utilized it to propose a simplified prediction model with features selected by XGB and LASSO algorithms. The high interpretability and promising performance of the simplified model make it suitable to be applied. Since the present study is an initial attempt, future studies will investigate the extensibility of advanced approaches from other domains^48^ and improvement of the existing algorithms^49,50^ in predicting the persistence of AKI.

Our study has important clinical significance. The prediction model for persistent AKI can assist risk stratification and therapeutic strategies of SA-AKI patients at an early stage^9^. For high-risk patients, large fluid infusion should be cautious to avert detrimental fluid overload. The requirement and optimal timing of RRT can be evaluated for patients without the indication of urgent hemodialysis. Constant monitoring is necessary, especially for high-risk patients, to assess the hemodynamic and fluid status, kidney function, complications of AKI and the risk of long-term adverse sequelae. Additionally, high-risk patients may be the ideal population for AKI clinical trials because they tend to experience no spontaneous and rapid reversal of AKI.

Many factors, including demographics, comorbidities and disease severity, can affect short-term renal recovery^51^. In this study, fourteen predictors of persistent AKI were identified by XGB and LASSO algorithms. The SCr and UO criteria of AKI stage were both strong predictors of persistent AKI. The results further supported that patients who meet both the SCr and UO criteria for AKI are at higher risk of death or RRT^52^. Among patient-related variables, age, CKD, diabetes mellitus and congestive heart failure were identified as predictors of persistent AKI, as they may cause reduced glomerular reserve and delayed or incomplete renal recovery^51^. During sepsis, systemic disease status and distant organ dysfunction may affect the evolution of AKI^53^. Recent studies have suggested that acute respiratory distress syndrome is associated with a strong trend toward developing AKI^54,55^. A close relationship between mechanical ventilation and worsening of renal function was observed in a large cohort of ICU patients^56^. Metabolic acidosis is common in SA-AKI patients and can directly influence cardiac contractility and sensitivity of adrenergic receptors^57^. Coagulopathy, mainly caused by the activation or injury of endothelial cells, plays an important role in the pathogenesis of SA-AKI through microcirculatory dysfunction^58^. Our results further demonstrated that sepsis-related factors, including those relevant to respiratory failure, metabolic acidosis and coagulation disorder, could contribute to the prediction of persistent AKI. Further studies are required to investigate the exact pathophysiological mechanisms of reversibility of SA-AKI and determine whether modification of these factors can facilitate renal recovery and improve prognosis.

There are some strengths of our study. Firstly, with the combination of logistic regression and feature selection by machine learning algorithms, we established a simplified risk prediction model with high practicability and interpretability. Secondly, fourteen predictors of persistent AKI were selected by state-of-the-art algorithms. The unbiased machine learning methods can help identify important features, which are clinically significant but may be ignored by clinicians according to their traditional experience. Thirdly, an easy-to-use risk calculator was developed to allow automatic quantified assessment of the risk of persistent AKI, which is a useful tool for clinicians to identify high-risk patients and improve clinical decision-making abilities.

However, this study is also subject to some limitations. Firstly, it was a single-center retrospective study based on a publicly accessible database, which may limit the generalizability of the prediction model in patients with differently distributed features. External validation is still necessary, and clinical impact studies should be conducted to assess the model’s effectiveness before its clinical implementation. Secondly, although we only included variables with ≤ 30% missing values, there were still 2.2% of all observations missing. Some candidate variables were excluded owing to a large percentage of missing values. Finally, similar to other machine learning models, the performance of our model was not perfect^38,45,47^. Possible reasons include the limited set of predictors, retrospective study design and heterogeneity of SA-AKI patients. Novel biomarkers, which were potential predictors of persistent AKI but not routinely measured in clinical settings, were not included in the prediction model. Based on this study, there is a continuing need for future studies to combine the clinical prediction model and biomarkers to predict persistent AKI.

In conclusion, machine learning algorithms are helpful to distinguish between transient and persistent AKI and identify the predictors of persistent AKI in critically ill septic patients. A simplified 14-variable risk prediction model was developed and validated with high practicability and interpretability. A risk calculator was established to facilitate its widespread application in daily clinical practice, which may help identify high-risk patients, guide treatment decisions and improve prognosis. Future prospective studies are needed to demonstrate the model’s generalizability and effectiveness and determine whether the addition of novel biomarkers could improve the predictive ability.

## Supplementary Information

Supplementary Information.

## Data Availability

The datasets analyzed during the current study are available in the MIMIC-III database (https://mimic.physionet.org/).
