# Supplementary material for: Machine learning for early discrimination between transient and persistent acute kidney injury in critically ill patients with sepsis
Source: Sci Rep. 2021 Oct 12;11:20269. doi: 10.1038/s41598-021-99840-6 (PMC8511088; doi:10.1038/s41598-021-99840-6)

Machine learning for early discrimination between transient and persistent acute kidney injury in critically ill patients with sepsis

Xiao-Qin Luo[1], Ping Yan[1], Ning-Ya Zhang[2], Bei Luo[3], Mei Wang[1], Ying-Hao Deng[1], Ting Wu[1], Xi Wu[1], Qian Liu[1], Hong-Shen Wang[1], Lin Wang[1], Yi-Xin Kang[1] & Shao-Bin Duan[1*]

[1]Department of Nephrology, The Second Xiangya Hospital of Central South University, Hunan Key Laboratory of Kidney Disease and Blood Purification, Changsha 410011, Hunan, China

[2]Information Center, The Second Xiangya Hospital of Central South University, Changsha 410011, Hunan, China

[3]Department of Information Systems, City University of Hong Kong; Tat Chee Avenue, Kowloon Hong Kong SAR 999077, China

***Corresponding author:** Professor Shao-Bin Duan, The Second Xiangya Hospital of Central South University, 139 Renmin Road, Changsha 410011, Hunan, China. E-mail: duansb528@csu.edu.cn

Table S1. Missing data of baseline characteristics.

Variables	Missing data, n (%)
Age (year)	0 (0.0)
Sex, male	0 (0.0)
Ethnicity	0 (0.0)
ICU type	0 (0.0)
Admission type	0 (0.0)
Comorbidities	
Hypertension	0 (0.0)
Diabetes mellitus	0 (0.0)
Congestive heart failure	0 (0.0)
Peripheral vascular disease	0 (0.0)
Chronic pulmonary disease	0 (0.0)
Liver disease	0 (0.0)
AIDS	0 (0.0)
Metastatic cancer	0 (0.0)
Chronic kidney disease	0 (0.0)
Vital signs	
Minimum temperature (℃)	149 (2.5)
Maximum temperature (℃)	149 (2.5)
Maximum heart rate (bpm)	9 (0.2)
Maximum respiratory rate (bpm)	9 (0.2)
Minimum MAP (mmHg)	9 (0.2)
Laboratory data	
Minimum hemoglobin (g/dL)	4 (0.07)
Minimum WBC ($\times 10^9$/L)	3 (0.05)
Maximum WBC ($\times 10^9$/L)	3 (0.05)
Minimum platelet ($\times 10^9$/L)	3 (0.05)
Maximum bilirubin (mg/dL)	2552 (42.6)
Minimum albumin (g/dL)	3150 (52.6)
Minimum pH	824 (13.8)
Minimum PaO_2 (mmHg)	824 (13.8)
Minimum $PaCO_2$ (mmHg)	824 (13.8)
Maximum $PaCO_2$ (mmHg)	824 (13.8)
Maximum anion gap (mmol/L)	13 (0.2)
Minimum sodium (mmol/L)	2 (0.03)
Maximum sodium (mmol/L)	2 (0.03)
Maximum potassium (mmol/L)	2 (0.03)
Minimum chloride (mmol/L)	2 (0.03)
Maximum chloride (mmol/L)	2 (0.03)
Minimum bicarbonate (mmol/L)	2 (0.03)
Maximum lactate (mmol/L)	1309 (21.9)
Maximum INR	408 (6.8)
Maximum PTT (sec)	428 (7.2)
Interventions	
Mechanical ventilation	0 (0.0)
Vasopressors	0 (0.0)
Diuretics	0 (0.0)
RRT initiation	0 (0.0)

Daily fluid infusion (mL)	76 (1.3)
AKI stage by SCr criteria	0 (0.0)
AKI stage by UO criteria	0 (0.0)

ICU, intensive care unit; AIDS, acquired immune deficiency syndrome; MAP, mean arterial pressure; WBC, white blood cell; PaO_2, partial pressure of oxygen; $PaCO_2$, partial pressure of carbon dioxide; INR, international normalized ratio; PTT, partial thromboplastin time; RRT, renal replacement therapy; AKI, acute kidney injury; SCr, serum creatinine; UO, urine output.

Table S2. List of variables included in the machine learning models.

Variable	Type	Unit
Age	Continuous	year
Sex	Categorical	-
Ethnicity	Categorical	-
ICU type	Categorical	-
Admission type	Categorical	-
Hypertension	Categorical	-
Diabetes mellitus	Categorical	-
Congestive heart failure	Categorical	-
Peripheral vascular disease	Categorical	-
Chronic pulmonary disease	Categorical	-
Liver disease	Categorical	-
AIDS	Categorical	-
Metastatic cancer	Categorical	-
Chronic kidney disease	Categorical	-
Minimum temperature	Continuous	℃
Maximum temperature	Continuous	℃
Maximum heart rate	Continuous	bpm
Maximum respiratory rate	Continuous	bpm
Minimum MAP	Continuous	mmHg
Minimum hemoglobin	Continuous	g/dL
Minimum WBC	Continuous	$\times 10^9$/L
Maximum WBC	Continuous	$\times 10^9$/L
Minimum platelet	Continuous	$\times 10^9$/L
Minimum pH	Continuous	-
Minimum PaO_2	Continuous	mmHg
Minimum $PaCO_2$	Continuous	mmHg
Maximum $PaCO_2$	Continuous	mmHg
Maximum anion gap	Continuous	mmol/L
Minimum sodium	Continuous	mmol/L
Maximum sodium	Continuous	mmol/L
Maximum potassium	Continuous	mmol/L
Minimum chloride	Continuous	mmol/L
Maximum chloride	Continuous	mmol/L
Minimum bicarbonate	Continuous	mmol/L
Maximum lactate	Continuous	mmol/L
Maximum INR	Continuous	-
Maximum PTT	Continuous	sec
Mechanical ventilation	Categorical	-
Vasopressors	Categorical	-
RRT initiation	Categorical	-
Diuretics	Categorical	-
Daily fluid infusion	Continuous	mL
AKI stage by SCr criteria	Categorical	-
AKI stage by UO criteria	Categorical	-

ICU, intensive care unit; AIDS, acquired immune deficiency syndrome; MAP, mean arterial pressure; WBC, white blood cell; PaO_2, partial pressure of oxygen; $PaCO_2$, partial pressure of carbon dioxide; INR,

international normalized ratio; PTT, partial thromboplastin time; RRT, renal replacement therapy; AKI, acute kidney injury; SCr, serum creatinine; UO, urine output.

Table S3. Locations of culture-positive specimens in patients stratified by the persistence of AKI.

Specimen	Transient AKI (n=2179)	Persistent AKI (n=3805)	*P* value
Sputum, n (%)	248 (11.4)	504 (13.2)	0.040
Urine, n (%)	255 (11.7)	500 (13.1)	0.12
Blood, n (%)	205 (9.4)	394 (10.4)	0.26
Bronchoalveolar lavage or bronchial washings, n (%)	48 (2.2)	87 (2.3)	0.91
Tissue, n (%)	30 (1.4)	66 (1.7)	0.34
Abscess, n (%)	11 (0.5)	15 (0.4)	0.67
Stool, n (%)	17 (0.8)	34 (0.9)	0.75
Peritoneal fluid, n (%)	7 (0.3)	33 (0.9)	0.02
Pleural fluid, n (%)	6 (0.3)	19 (0.5)	0.28
Bile, n (%)	13 (0.6)	13 (0.3)	0.22
Other, n (%)	209 (9.6)	394 (10.4)	0.37

AKI, acute kidney injury.

Table S4. Pathogenic microorganisms in patients stratified by the persistence of AKI.

Pathogens	Transient AKI (n=2179)	Persistent AKI (n=3805)	*P* value
Gram-positive bacteria, n (%)			
Staphylococcus	314 (14.4)	614 (16.1)	0.08
Enterococcus	78 (3.6)	184 (4.8)	0.026
Streptococcus	93 (4.3)	168 (4.4)	0.84
Corynebacterium	22 (1.0)	48 (1.3)	0.46
Clostridium	25 (1.2)	44 (1.2)	1.00
Lactobacillus	6 (0.3)	14 (0.4)	0.72
Other	50 (2.3)	101 (2.7)	0.44
Gram-negative bacteria, n (%)			
Escherichia coli	117 (5.4)	201 (5.3)	0.93
Pseudomonas aeruginosa	43 (2.0)	81 (2.1)	0.76
Klebsiella	52 (2.4)	103 (2.7)	0.51
Proteus	28 (1.3)	35 (0.9)	0.23
Enterobacter	11 (0.5)	22 (0.6)	0.85
Serratia	9 (0.4)	13 (0.3)	0.83
Haemophilus	19 (0.9)	26 (0.7)	0.51
Bacteroides fragilis	18 (0.8)	41 (1.1)	0.42
Stenotrophomonas maltophilia	3 (0.1)	10 (0.3)	0.40
Acinetobacter	8 (0.4)	7 (0.2)	0.27
Other	91 (4.2)	194 (5.1)	0.12
Fungi, n (%)			
Yeast	149 (6.8)	369 (9.7)	<0.001
Candida	26 (1.2)	53 (1.4)	0.59
Aspergillus	5 (0.2)	17 (0.5)	0.27
Other	16 (0.7)	21 (0.6)	0.49
Virus, n (%)	8 (0.4)	17 (0.5)	0.80

AKI, acute kidney injury.

Table S5. The 20 most common diagnoses in SA-AKI patients.

ICD-9 codes	Description	n (%)
The 20 most common diagnoses in patients with transient AKI		
4019	Unspecified essential hypertension	1008 (46.3)
42731	Atrial fibrillation	661 (30.3)
4280	Congestive heart failure, unspecified	650 (29.8)
51881	Acute respiratory failure	549 (25.2)
41401	Coronary atherosclerosis of native coronary artery	535 (24.6)
5849	Acute kidney failure, unspecified	505 (23.2)
2724	Other and unspecified hyperlipidemia	493 (22.6)
25000	Diabetes mellitus without mention of complication, type II or unspecified type, not stated as uncontrolled	393 (18.0)
5990	Urinary tract infection, site not specified	369 (16.9)
2859	Anemia, unspecified	323 (14.8)
99592	Severe sepsis	318 (14.6)
486	Pneumonia, organism unspecified	294 (13.5)
53081	Esophageal reflux	293 (13.4)
5070	Pneumonitis due to inhalation of food or vomitus	291 (13.4)
0389	Unspecified septicemia	282 (12.9)
2449	Unspecified acquired hypothyroidism	240 (11.0)
2851	Acute posthemorrhagic anemia	233 (10.7)
2720	Pure hypercholesterolemia	218 (10.0)
2762	Acidosis	210 (9.6)
78552	Septic shock	207 (9.5)
The 20 most common diagnoses in patients with persistent AKI		
4019	Unspecified essential hypertension	1580 (41.5)
51881	Acute respiratory failure	1463 (38.4)
42731	Atrial fibrillation	1446 (38.0)
4280	Congestive heart failure, unspecified	1438 (37.8)
5849	Acute kidney failure, unspecified	1253 (32.9)
41401	Coronary atherosclerosis of native coronary artery	892 (23.4)
25000	Diabetes mellitus without mention of complication, type II or unspecified type, not stated as uncontrolled	882 (23.2)
99592	Severe sepsis	854 (22.4)
5845	Acute kidney failure with lesion of tubular necrosis	727 (19.1)
2724	Other and unspecified hyperlipidemia	709 (18.6)
5990	Urinary tract infection, site not specified	708 (18.6)
486	Pneumonia, organism unspecified	668 (17.6)
0389	Unspecified septicemia	667 (17.5)
2762	Acidosis	665 (17.5)
5070	Pneumonitis due to inhalation of food or vomitus	598 (15.7)
78552	Septic shock	581 (15.3)
2859	Anemia, unspecified	560 (14.7)
2875	Thrombocytopenia, unspecified	488 (12.8)
2851	Acute posthemorrhagic anemia	464 (12.2)
2760	Hyperosmolality and/or hypernatremia	428 (11.2)

SA-AKI, sepsis-associated acute kidney injury; AKI, acute kidney injury. ICD-9, International Classification of Diseases 9th Edition.

Table S6. Baseline characteristics of patients in the training and testing set.

Variables	Training set (n=4188)	Testing set (n=1796)	*P* value
Age (year)	69 (57-80)	69 (56-80)	0.20
Sex, male, n (%)	2339 (55.9)	1016 (56.6)	0.63
Ethnicity, n (%)			0.91
White	3129 (74.7)	1336 (74.4)	
Black	302 (7.2)	135 (7.5)	
Other	757 (18.1)	325 (18.1)	
ICU type, n (%)			0.46
MICU	1662 (39.7)	741 (41.3)	
SICU/TSICU	1191 (28.4)	507 (28.2)	
CCU/CSRU	1335 (31.9)	548 (30.5)	
Admission type, n (%)			0.53
Elective	595 (14.2)	266 (14.8)	
Emergency	3504 (83.7)	1485 (82.7)	
Urgent	89 (2.1)	45 (2.5)	
Comorbidities, n (%)			
Hypertension	2303 (55.0)	994 (55.3)	0.82
Diabetes mellitus	1235 (29.5)	550 (30.6)	0.40
Congestive heart failure	1596 (38.1)	653 (36.4)	0.21
Peripheral vascular disease	575 (13.7)	242 (13.5)	0.82
Chronic pulmonary disease	923 (22.0)	388 (21.6)	0.73
Liver disease	449 (10.7)	210 (11.7)	0.29
AIDS	37 (0.9)	20 (1.1)	0.49
Metastatic cancer	237 (5.7)	106 (5.9)	0.76
Chronic kidney disease	563 (13.4)	242 (13.5)	1.00
Vital signs			
Minimum temperature (℃)	35.9 (35.5-36.4)	35.9 (35.6-36.4)	0.43
Maximum temperature (℃)	37.8 (37.3-38.4)	37.8 (37.3-38.4)	0.71
Maximum heart rate (bpm)	112 (98-128)	113 (99-129)	0.31
Maximum respiratory rate (bpm)	30 (26-35)	30 (26-34)	0.22
Minimum MAP (mmHg)	53 (47-59)	53 (47-59)	0.56
Laboratory data			
Minimum hemoglobin (g/dL)	9.1 (8.1-10.4)	9.1 (8.1-10.3)	0.49
Minimum WBC ($\times 10^9$/L)	9.6 (6.9-12.9)	9.7 (6.9-13.2)	0.57
Maximum WBC ($\times 10^9$/L)	14.4 (10.6-19.3)	14.2 (10.5-19.2)	0.40
Minimum platelet ($\times 10^9$/L)	152 (101-220)	153 (99-225)	0.56
Minimum pH	7.33 (7.26-7.38)	7.33 (7.26-7.38)	0.23
Minimum PaO_2 (mmHg)	81 (67-100)	82 (67-101)	0.59
Minimum $PaCO_2$ (mmHg)	34 (30-38)	34 (30-39)	0.41
Maximum $PaCO_2$ (mmHg)	46 (40-53)	45 (40-53)	0.32
Maximum anion gap (mmol/L)	15 (13-18)	15 (13-18)	0.58
Minimum sodium (mmol/L)	136 (133-139)	136 (133-139)	0.66
Maximum sodium (mmol/L)	141 (138-143)	140 (138-143)	0.63
Maximum potassium (mmol/L)	4.6 (4.2-5.1)	4.6 (4.2-5.1)	0.06
Minimum chloride (mmol/L)	103 (100-107)	103 (100-107)	0.73
Maximum chloride (mmol/L)	109 (105-112)	109 (105-112)	0.35
Minimum bicarbonate (mmol/L)	21 (18-24)	21 (18-24)	0.23
Maximum lactate (mmol/L)	2.3 (1.7-3.4)	2.2 (1.6-3.3)	0.34

Maximum INR	1.4 (1.3-1.9)	1.5 (1.3-1.9)	0.20
Maximum PTT (sec)	38.6 (30.5-57.4)	38.7 (30.5-57.9)	0.80
Interventions			
Mechanical ventilation, n (%)	3186 (76.1)	1339 (74.6)	0.22
Vasopressors, n (%)	2266 (54.1)	948 (52.8)	0.36
RRT initiation, n (%)	171 (4.1)	86 (4.8)	0.24
Diuretics, n (%)	2218 (53.0)	962 (53.6)	0.69
Daily fluid infusion (mL)	3097 (1932-4558)	3019 (1921-4596)	0.66
AKI stage by SCr criteria, n (%)			0.63
1	1229 (29.3)	556 (31.0)	
2	282 (6.7)	123 (6.9)	
3	332 (7.9)	140 (7.8)	
AKI stage by UO criteria, n (%)			0.11
1	626 (14.9)	281 (15.6)	
2	2108 (50.3)	854 (47.6)	
3	906 (21.6)	433 (24.1)	
Persistent AKI	2644 (63.1)	1161 (64.6)	0.28

ICU, intensive care unit; MICU, medical intensive care unit; SICU, surgical intensive care unit; TSICU, trauma surgical intensive care unit; CCU, coronary care unit; CSRU, cardiac surgery recovery unit; AIDS, acquired immune deficiency syndrome; MAP, mean arterial pressure; WBC, white blood cell; PaO_2, partial pressure of oxygen; $PaCO_2$, partial pressure of carbon dioxide; INR, International normalized ratio; PTT, partial thromboplastin time; RRT, renal replacement therapy; AKI, acute kidney injury; SCr, serum creatinine; UO, urine output.

Continuous variables were presented as median (interquartile range) and categorical variables were presented as n (%).

Figure S1. Risk calculator for the persistence of SA-AKI.

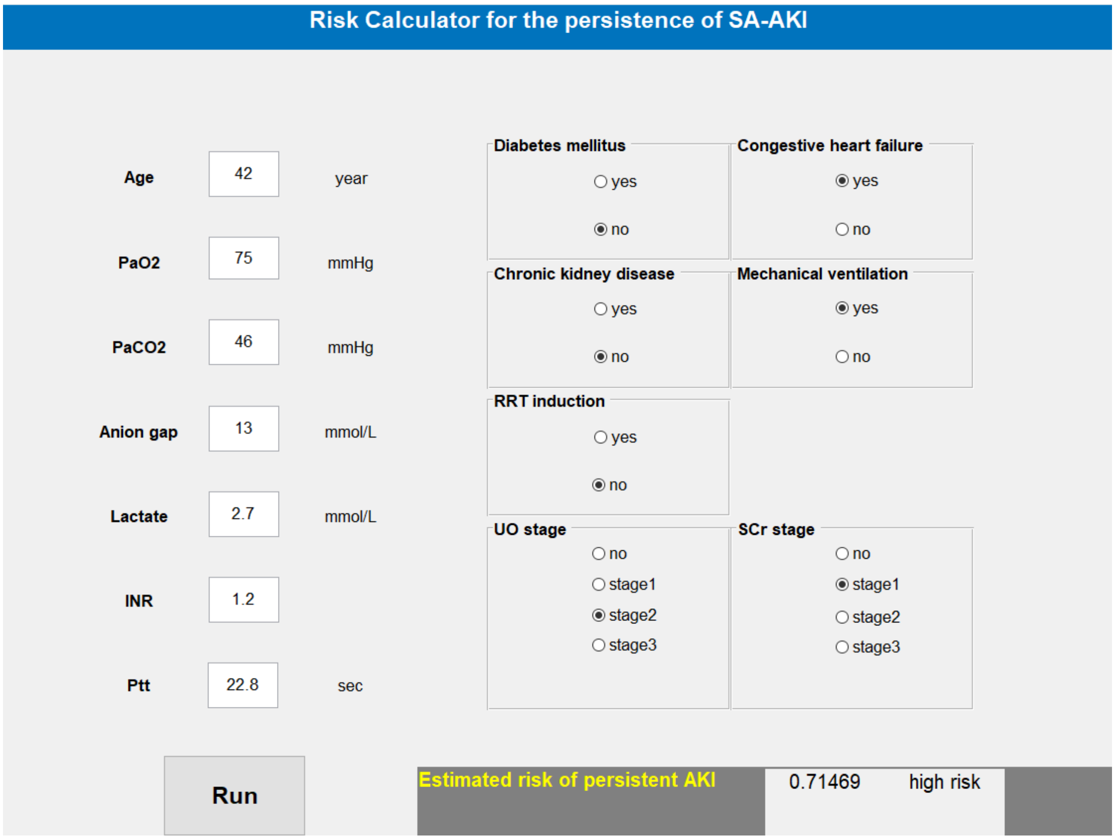

Supplement: Supplementary file 1 — Supplementary Information. [file 41598_2021_99840_MOESM1_ESM.pdf]
